# Mini review: Prevention of mother–child transmission of HIV: 25 years of continuous progress toward the eradication of pediatric AIDS?

**DOI:** 10.1080/21505594.2019.1697136

**Published:** 2019-12-29

**Authors:** Stéphane Blanche

**Affiliations:** Pediatric Immunology-Hematology Unit, Hôpital Necker–Enfants Malades, Assistance Publique-Hôpitaux de Paris (AP-HP) and Faculté de Médecine Paris Descartes, Paris, France

**Keywords:** HIV, pregnancy, children, antiretroviral, prophylaxis

## Abstract

Prevention of mother-to-child transmission with antiretrovirals is extraordinarily effective. When medically well followed, a mother living with human immunodeficiency virus can now expect to avoid transmitting the virus to her child. Despite the immense difficulties inherent in the global implementation of this treatment, the virtual disappearance of pediatric AIDS can be considered in the long term.

## Introduction

The possibility of transmission of the human immunodeficiency virus (HIV) from mother to child was identified very early after the onset of the epidemic, as early as 1983 [1]. The infection of the child is characterized by a high risk of early and severe evolution with encephalopathy and death in the first 2 y of life [2]. The mechanism of HIV transmission to the child during pregnancy is still hypothetical, based on indirect arguments, and probably not unequivocal. Without intervention, 15–20% of non-breastfed newborns are infected [3]; however, only one-third of the infected children has detectable virus at birth, demonstrating that viral replication started *in utero*, as opposed to infected children, whose virus becomes detectable only after a few weeks of life. By analogy with the dynamics of the appearance of the virus during primary infection of the adult, it is possible that contamination in the other two-thirds of infected newborns occurs *peri-partum*. The placenta, therefore, appears to be largely protective, given the minority of children infected *in utero* [4]. Most placental histopathological studies do not find specific lesions in HIV-infected women, in contrast to many other pathogens [4–7]. There is, however, an increased risk of chorioamnionitis, but the association with the risk of transmission of the virus is still debated [4]. The virus can be identified in the placenta, and phylogenetic studies between placental behavior and the blood compartment prove that an independent cycle of replication takes place. In addition to the maternal CD4 lymphocytes that it infects, the virus can be isolated from syncytiotrophoblasts and extra-vilotic mononuclear cells, especially fetal macrophages and Hofbauer cells [4,8]. These cells have receptors and co-receptors (CD4, CCR5, CX R4, CD209) that allow entry of the virus, as well as Fcγ receptors capable of capturing virion–antibody complexes. The quantity of virus in placental tissue is, however, low [8]. Remarkably, many studies have shown that Hofbauer cells can limit viral replication through regulatory cytokine production [9]. In situations of high maternal viral blood replication, it is possible that this protective mechanism is overwhelmed, allowing passage of the virion to the free or intracellular state in the fetal circulation. Co-factors can then very likely intervene to facilitate or inhibit transmission to the child; the presence of maternal co-infection with microorganisms, such as plasmodium, mycobacterium tuberculosis, or Cytomegalovirus (CMV), is associated with a higher risk of transmission. For example, cytomegalovirus is well known for inducing placental inflammation, which may increase the number of HIV-responsive cells. Conversely, maternal neutralizing antibodies passively transmitted to the child [10], as well as a genetic profile of relative resistance to the child’s HIV infection, may protect him [11]. Events leading to infection of the child during childbirth are of another order. The risk linked to the sometimes-traumatic passage through the vagina in contact with secretions and maternal blood was first evoked. The (partial) protective effect of cesarean section initially supported this hypothesis until it was shown that only cesareans performed prior to the onset of labor had a protective effect. The phenomenon of transplacental micro-transfusion from mother to child that occurs during labor, well known in perinatal medicine, is probably the main source of *peri-partum* infection. Elegant work based on the Human Leucocytes Antigens (HLA) homology between mother and child and the risk of HIV transmission to the child indirectly reinforces this hypothesis; greater homology would reduce allo-reactivity of the child against maternal cells and therefore their persistence and the risk of viral transmission [12]. The diagnosis of infection by direct identification of the virus then requires a few weeks to be detectable by RT-PCR of the RNA (free virus) or DNA (intracellular DNA virus). The sensitivity of these two techniques is almost 100% from the age of 3 months if the child is not exposed to post-natal breastfeeding [13]. If breastfed, the final diagnosis of non-infection is performed by PCR 2 to 3 weeks after weaning. Transmission through breastfeeding is transmucosal for free and intracellular virus and facilitated by the possible epithelial inflammation induced by mixed breastfeeding. The risk depends on the duration of breastfeeding but appears to be higher during the initial period because colostrum is rich in maternal immune cells [14].

Overall, the major risk factor of infection of the child *pre-, per-, or post-partum* is the level of maternal viral replication, precisely quantifiable by plasma RT-PCR. The risk of *in utero* transmission is higher if there is a very high maternal viral load or if the mother’s primo infection occurs during pregnancy [15].

By 1994, 10 y after the first descriptions of infected children, the epidemic was progressing inexorably, and its catastrophic scale in sub-Saharan Africa was evident. At the same time, the first monotherapy treatments using nucleoside analogs proved to be very disappointing and only marginally influenced the course of the disease. It is in this very pessimistic context that the interim analysis of a placebo-controlled US–French protocol to prevent transmission during pregnancy by zidovudine (ACTG076-ANRS024) provided the first stunning success of antiretroviral therapy: oral zidovudine administered *prepartum*, intravenously during labor, and then *postpartum* in the child reduced the risk of transmission to the child by two-thirds [16]. This unexpected result led to the immediate interruption of the trial and opened a new era of hope that has been growing since through the exceptional mobilization of institutes, associations, and individuals. Twenty-five years later, it is possible to seriously consider the unimaginable: the possible eradication of pediatric AIDS [17].

## Prevention of mother-to-child transmission with antiretrovirals

**Figure 1.**
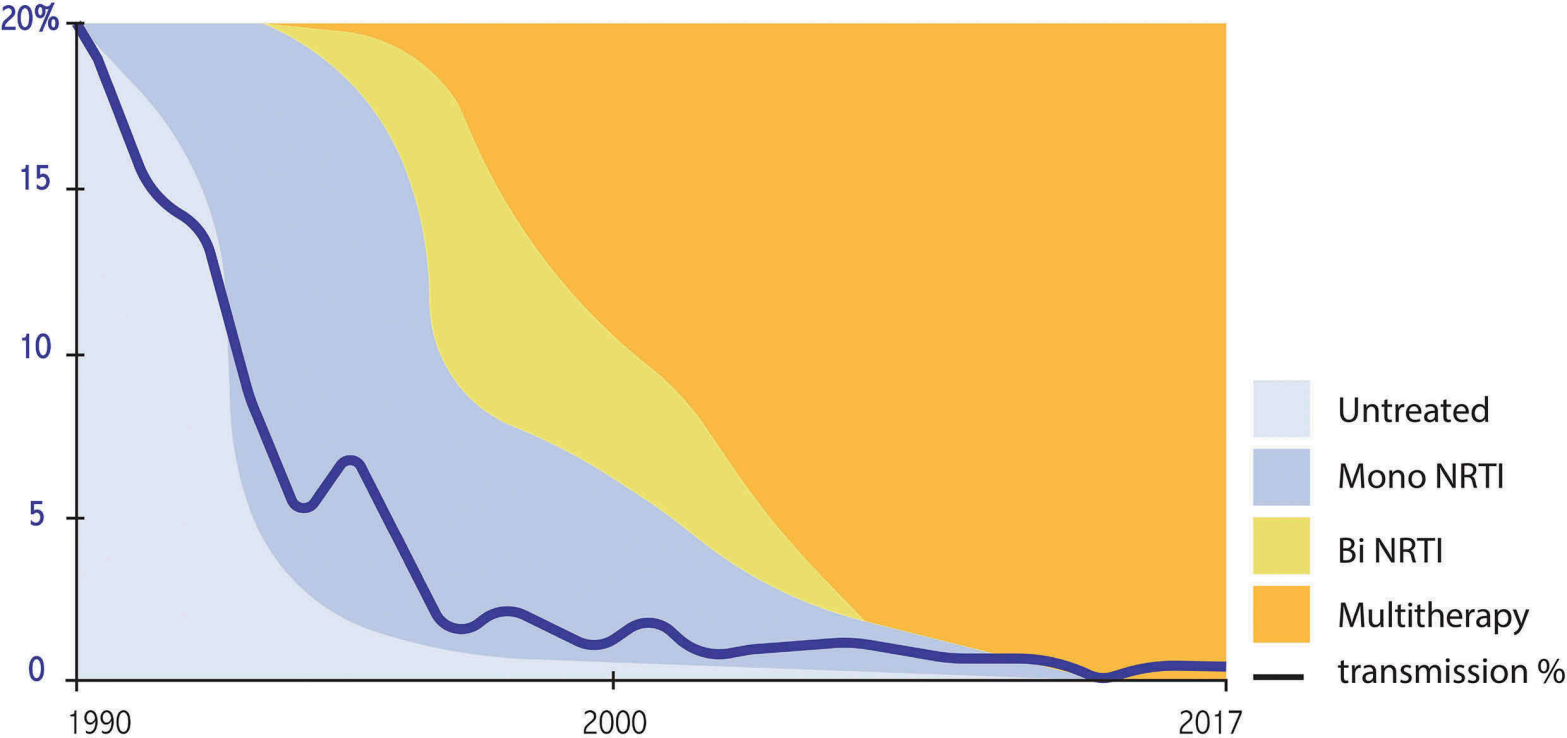
Evolution of the risk of transmission to the child depending on the intensity of antiretroviral treatment in mono, bi, or triple therapy. French Perinatal Study 1985-2017 (unpublished). NRTI: nucleos(t)ide reverserse transcriptase inhibitor.

Soon after, it was demonstrated that bi (1995) and then triple (1996) antiretroviral therapy in HIV-infected adults allows efficient and lasting inhibition of viral replication, followed by a major immunological and clinical benefit [18]. The close link between the level of viral replication and the risk of transmission to the child logically justified applying such therapeutic escalation to pregnant women; the infection rate steadily dropped to near zero with the use of three molecules, with an undetectable viral load at delivery (Figure 1). Although there is an overall transmission rate of less than 1%, there is still a significant risk gradient depending on the duration of treatment during pregnancy (Table 1). Treatment started before pregnancy, with undetectable viral replication up to birth, is even associated with a zero risk of transmission, a finding now validated in several cohorts totaling several thousand women [19]. These extraordinary results – unimaginable in the darkest hours of the epidemic – have completely overshadowed the interest in planned cesareans and demonstrated to be effective before the era of antiretroviral combinations. They also made it possible to abandon intensification during labor, initially proposed in the form of a continuous infusion of zidovudine. The post-natal phase, administered to newborns, is currently maintained, although it is not known whether it is necessary if the mother was properly treated during pregnancy. In 2019, the preventive strategy in previously untreated women is still empirically based on a combination comprising two nucleoside analogs and a third molecule of one of the other classes: antiprotease, non-nucleoside reverse-transcriptase inhibitor, or integrase strand-transfer inhibitor. Alternatives have been proposed if there is resistance or intolerance (rare) to one or more of these molecules [20–22]. In a pragmatic approach, the general recommendation for women who have been effectively treated before pregnancy is to maintain the same treatment. This sometimes leads to the continuation of new molecules during pregnancy for which the toxicity data are still limited or virtually zero. The recent warning about dolutegravir (see below), the latest generation molecule, calls for caution in a context in which maximum preventive efficacy can be obtained with older molecules [23]. In contrast, intensification strategies are proposed if the mother has been insufficiently treated: the addition of a fourth molecule at the end of pregnancy, performance of a cesarean section before labor, perfusion of zidovudine, or intensification of the treatment of the newborn with bi or even triple therapy [24]. These various measures make it possible to drastically reduce the transmission rate, even if there was insufficient treatment during pregnancy. Such prevention requires proactive attention from the medical teams: the earliest possible screening for maternal infection, optimization of the choice of maternal treatment depending on possible prior resistance, assistance in taking the medication, reactivity if there is an insufficient virological result, and anticipation of the choice of treatment for the newborn. The WHO and most countries issue regularly updated guidelines that should be consulted before prescribing antiretrovirals to pregnant women and newborns [20–22,25] (Table 2). Beyond purely therapeutic considerations, there is also the social and administrative vulnerability of many women living with HIV that can alter the overall prevention strategy. Maternal seroconversion during pregnancy after the initial negative screening is still one of the causes of failure, and it is necessary to be aware of the need to repeat the screen if a woman is considered to have a potentially high risk of infection during pregnancy.

**Table 1.** Residual transmission risk in mothers with undetectable viral load at delivery based on the timing of treatment initiation French Perinatal Study  (2000-2011).

Treatment start	*n*	% IC
Before conception	0/2651	0% [0.0–0.1]
1st trimester	1/507	0.2% [0.0–1.1]
2nd trimester	9/1735	0.5% [0.2–1.0]
3rd trimester	4/452	0.9% [0.2–2.3]

All women received a combination of at least three antiretroviral drugs.

Adapted from Ref. [19].

**Table 2.** General principles regarding the use of antiretroviral (ARV) drugs during pregnancy.

1. All pregnant women living with HIV should initiate ARV triple combination as early in pregnancy as possible, regardless of their plasma HIV RNA copy number or CD4 T lymphocyte count. *Earlier viral control is associated with lower risk of transmission* (see Table 1). An additional 4–6-week ﻿monotherapy is given to the neonate.
2. Maternal HIV viral load should be maintained below the limit of detection at all time points of pregnancy including antepartum and intrapartum as well as postnatally in case of breastfeeding to the neonate. Mother is encouraged to maintain the treatment after delivery even if the child is not breastfed.
3. Whenever possible, ARV drug-resistance genotype studies should be systematically performed before starting ARV drug regimens, including in women who are ARV naive, but treatment is initiated before results and adapted secondarily if necessary.
4. Previously treated women should continue their current regimens unless they include drugs known or suspected to be embryotoxic or new molecules for which there are no data on toxicity during pregnancy. Physicians are encouraged to visit updated guidelines. PK of some ARV changes in pregnancy; it may lead to lower plasma levels of drugs and necessitate increased dosages, more frequent dosing, boosting, or more frequent viral load monitoring.
5. In high-income countries, it is recommended to completely avoid breastfeeding, regardless of ART and maternal viral load. In resource-limited settings, maternal ARV treatment reduces the risk of transmission through breastfeeding.
ARV preferred choice (2019)
*Mother*: Two nucleoside reverse transcriptase inhibitors (NRTIs)^a^ associated with either a protease inhibitor (PI)^b^ or an integrase strand transfer inhibitor (INSTI)^c^. Alternative choices are possible. In countries with limited health resources, non-nucleodide reverse transcriptase inhibitor (NNRTI)^d^ as the third drug is still an option.
During delivery: NRTI^e^ perfusion, only if the maternal viral replication before delivery is not controlled. Pre-labor C section can also be discussed in this specific situation.
*Newborn*: NRTI^f^ or NNRTI^g^ monotherapy during 2–6 weeks. Dual or triple combination if untreated mother or in case of maternal uncontrolled viral replication at delivery (high risk of transmission)^h^.

^a^ABC: abacavir + 3TC: lamivudine or TDF: tenofovir + FTC: emtricitabine.

^b^rDRV: darunavir or rATZ: atazanavir, both boosted by ritonavir.

^c^RTG: raltegravir or DTG: dolutegravir (DTG is contraindicated during the first trimester of pregnancy).

^d^EFZ: efavirenz or NVP: nevirapine.

^e^Intravenous perfusion of AZT: zidovudine.

^f^AZT zidovudine.

^g^NVP nevirapine.

^h^AZT zidovudine + NVP nevirapine. Expert advice for triple therapy or in case of resistance on maternal viral isolate.

Adapted from Ref. [20–22].

## Worldwide expansion of prevention

The first success observed in Europe and North America in 1994 did not allow for the nihilism typical of highly endemic countries with often limited resources [26]. Almost all pregnant women with HIV live in sub-Saharan Africa, with their number estimated to be more than one million per year. Despite the complexity of the challenge, programs were rapidly put in place. Initially, simplified strategies were applied, such as the *peripartum* administration of a single dose of a non-nucleoside reverse-transcriptase inhibitor, nevirapine, and *postpartum* administration to children. This prophylaxis, although simplified to the extreme, reduced the risk of transmission by half but induced a particularly high rate of resistance in infected mothers and children. Remarkably, simplification of the preventive strategies was then abandoned, and they followed the European and North American recommendations [27]. The efficiency is intrinsically similar to that observed in the North, but two major obstacles have affected the effectiveness of such programs: the difficulty of screening pregnant women for HIV and the risk of post-natal contamination through breastfeeding.

### Difficulty of screening

**Figure 2.**
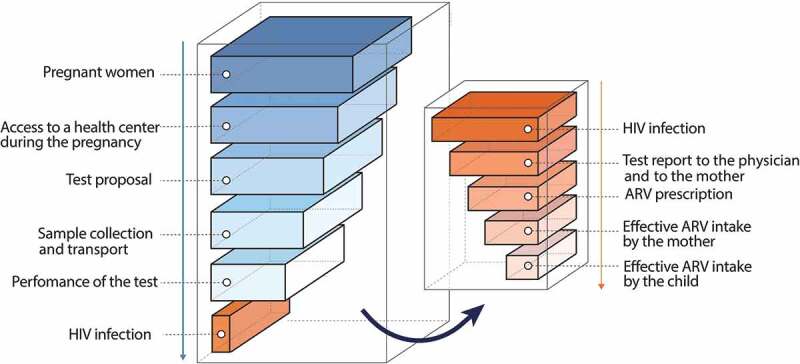
Theoretical cascade of “missed opportunities” for screening and treatment of pregnant women in low-resource countries.

**Figure 3.**
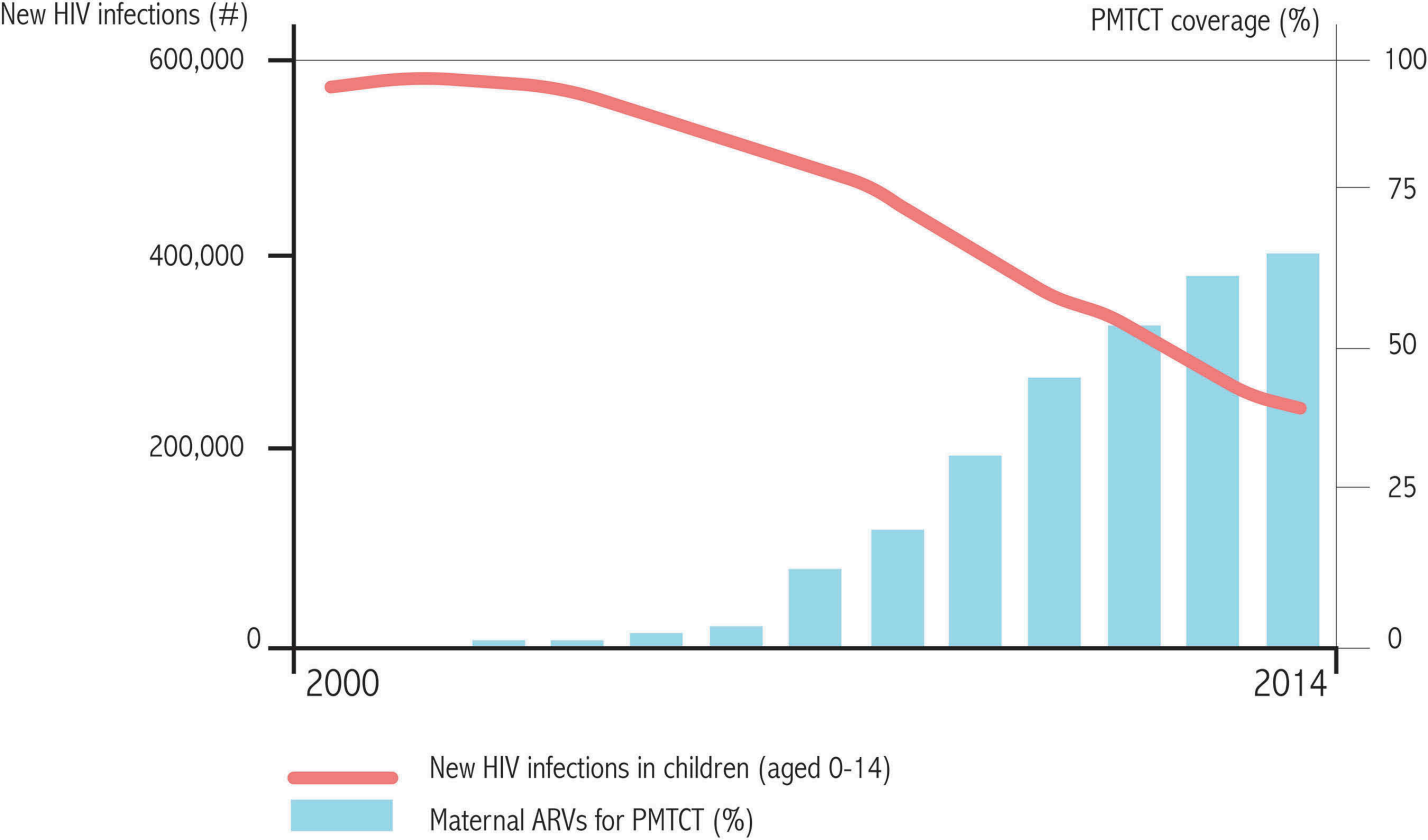
Evolution of the proportion of pregnant women tested for HIV-1 in 12 selected countries and evolution of the number of infected children. Source: UNAIDS [30].

The difficulties of screening pregnant women in sub-Saharan Africa are major. They are multifactorial (insufficient medical or paramedical time to propose, realize, and perform the tests, availability of facilities, maintenance of devices, etc.) leading to a “cascade of missed opportunity,” with a considerable loss of efficiency of programs in many locations (Figure 2). Beyond these organizational difficulties, screening is also difficult due to the sometimes-catastrophic family and social consequences of the stigmatization of women known to be infected with HIV. However, the situation is improving, and there has been a gradual increase in the number of women screened worldwide (Figure 3(a)), estimated by the WHO to be more than 65% in 2014, but with large regional disparities. It is possible that such progression is stalling and that the remaining 35% will be more difficult to reach: women who have no contact with a health structure during pregnancy, a high risk of stigma surrounding HIV, or the severe material deprivation of some rural areas of sub-Saharan Africa [28–30].

### Breastfeeding

The risk of post-natal transmission of HIV can ruin the effectiveness of *pre*- and *peripartum* prophylaxis. The first approach to address this issue was to try and shake up current dogma and consider artificial-feeding programs, despite their high cost, potential nutritional risks, and the risk of intestinal foodborne infection. The inability to apply this strategy at the national level became rapidly apparent [31]. Again, it was antiretrovirals that provided the solution by allowing “safe” breastfeeding. Two strategies are possible: treatment of the mother, rendering viral replication undetectable in milk, or treatment of the child, following the “post-exposure prophylaxis” approach. Remarkably, the preventive efficacy of these two approaches is similar, reducing the risk of transmission through breastfeeding to less than 1–2%, including during prolonged breastfeeding [14]. If the mother did not receive treatment before birth, the viral load in the milk becomes negative only after a few weeks of treatment. Protection is therefore not immediately effective, unlike “post-exposure prophylaxis,” which is effective from the first day of its administration to the child. Treatment given simultaneously to the child and the mother is certainly needed in this specific situation. Above all, maternal treatment – beyond the protection it provides to the child – provides long-term maternal health benefits, and this option (called B + in WHO programs) is now implemented in all countries in which breastfeeding is necessary [31]. Pregnancy thus appears to be a gateway to the long-term treatment of the mother and guarantor of her own health and that of her children. The great success of “safe breastfeeding” with antiretrovirals started a controversy about the end of restricting breastfeeding in high-income countries. However, although extremely reduced, zero risk has never been observed with safe breastfeeding, contrary to artificial feeding. Some authors have highlighted the specific risk of infection of the child with intracellular virus, despite the control of viral replication, as being able to cause cases of contamination of children while viral replication in milk was undetectable [32].

These components (large-scale screening and early and prolonged treatment with HAART during pregnancy and breastfeeding) have all substantially reduced the number of infected children worldwide. The 2014 estimate of infected children per year is approximately 200,000, down from more than 400,000 in the early 2000s (Figure 3) [30].

## The health of HIV-“exposed but non-infected” children

**Figure 4.**
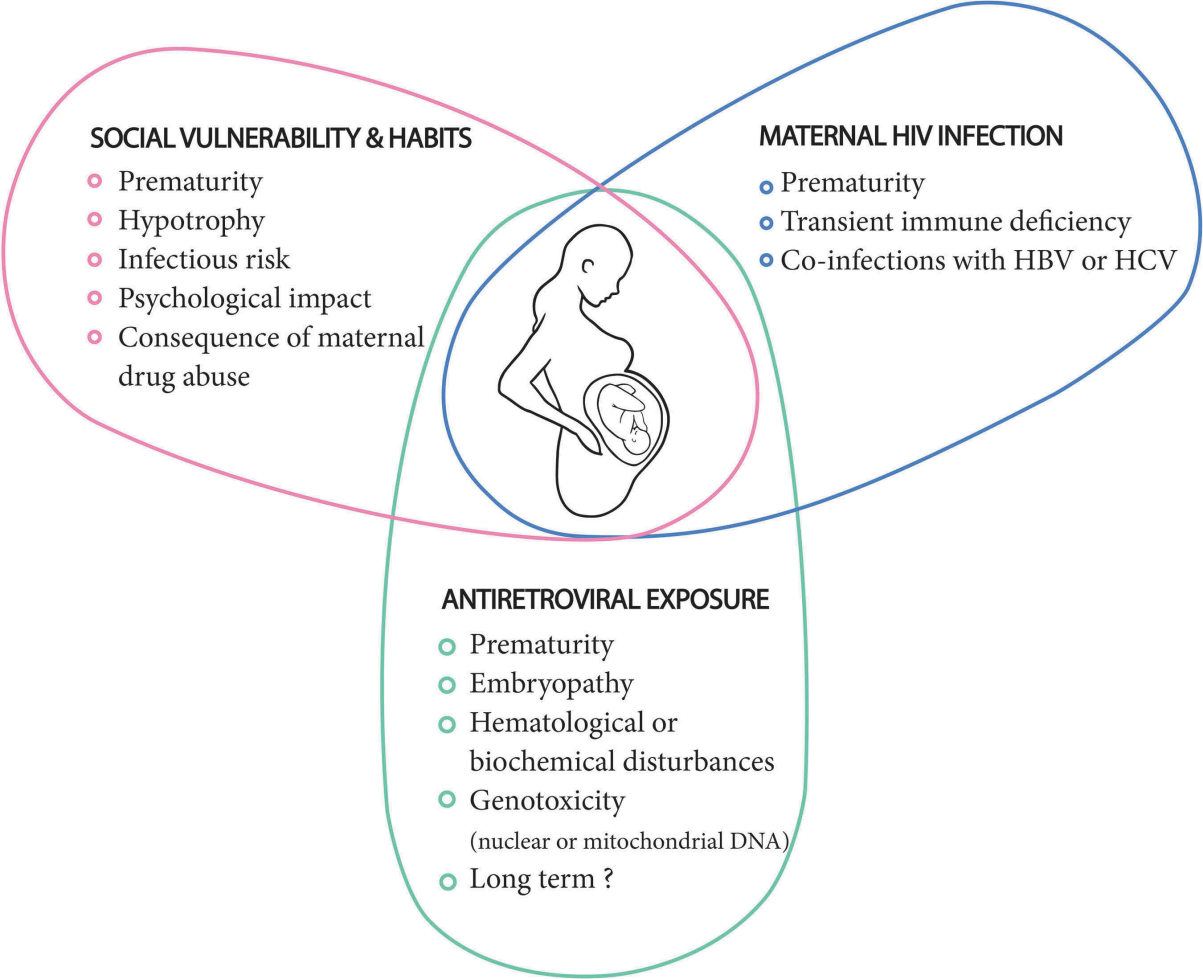
Health of exposed non-infected children. Triple potential risk.

More than one million children are born to HIV-infected mothers each year around the world. Even in the absence of any intervention, most children born to mothers infected with HIV are free of the infection. Now, almost all such children are HIV free due to the use of antiretrovirals. Although the health of most of these children is not of concern, a fine assessment is nonetheless necessary due to the low frequency of alerts and the number of children involved [33]. The health of these children is likely influenced by three factors, without it being easy to distinguish their respective roles (Figure 4). (1) The environment and maternal lifestyle can play an important role. Often linked to HIV infection, social vulnerability and poverty can lead to prematurity, fetal hypotrophy, and, in the post-natal period, undernutrition, increased risk of infections, and psychological consequences [34]. Maternal substance abuse, leading to HIV infection, can also have a serious impact on the child’s health, as already well established [35]. (2) The mother’s disease can potentially disrupt the progress of pregnancy, resulting in an increased risk of transmission of co-infections that sometimes accompany HIV infection (syphilis, *C. trachomatis*, *N. gonorrhea*, HBV, and HCV). It can also indirectly induce immune disturbances in children [36]. (3) Finally, antiretrovirals can potentially disrupt the course of pregnancy, induce embryonic malformations, or lead to organ toxicity, similar to that observed in adults and older children. Such toxicity may or may not be reversible and may be detectable from birth or later during the life of the child [37].

### Prematurity

Premature births to HIV-infected mothers were already recognized before the era of antiretrovirals, even in the absence of the usual risk factors for prematurity. The specific mechanisms associated with maternal HIV infection are not known. The severity of the immune deficiency increases such risk, but there is still a risk for women without significant immunodeficiency. It is possible that antiretrovirals in the class of protease inhibitors themselves induce paradoxical prematurity, despite improving maternal health, but the data are discordant [38]. Prematurity can have important consequences for the child, especially in countries in which access to neonatal care is limited or absent [39].

### Embryonic malformations

The malformation rate observed in children born to HIV-positive mothers is broadly similar to that observed in the general population [40]. Molecule-by-molecule analyses have identified some risk but with contradictory results depending on the study, such as those that followed the alert on efavirenz-related neural tube-closure abnormalities [39]. A low risk of cardiac malformation – most often of minimal severity – has been observed after exposure to zidovudine [40]. More recently, an alert has suggested possible neural-tube toxicity of dolutegravir, pending further analysis [23]. This information would urge caution in the large-scale use of this new molecule which has, however, much potential interest in terms of efficiency, genetic barriers to resistance, and cost.

### Biological disturbances observed at birth

All antiretroviral molecules – with the exception of the fusion inhibitor (T20, Fuzéon®) – pass the placenta to varying degrees [41], and the known pattern of toxicity of such molecules in the adult or child can be theoretically observed in the exposed newborn. Thus, reversible anemia has been linked to exposure to zidovudine, and hyper-bilirubinemia has been observed after exposure to atazanavir. Conversely, some toxicities are not, or only exceptionally, observed in exposed neonates, such as the hepatotoxicity of nevirapine, nephrotoxicity of tenofovir, or hyperlipidemia of lopinavir-r. However, the fetus may be more sensitive than adults to certain toxicities, such as mitochondrial dysfunction observed after exposure to zidovudine, didanosine, and stavudine (± lamivudine). Although such disturbances are most often asymptomatic, they can be at the origin of severe neurological syndromes and/or life-threatening hyperlactatemia [42].

### Increased risk of bacterial infections

Many cohorts in both low- and high-resource countries have shown an elevated incidence of bacterial infection in children born to HIV-positive mothers [43]. This risk mainly concerns encapsulated bacteria (*Hemophilus*, *Streptococcus*, *Pneumococcus*, *Bordetella pertussis*, etc.). A passive deficit in transplacental immunoglobulin has been well demonstrated. It mainly concerns children of women with severe immunodeficiency and lasts a few months, until the production of immunoglobulins by the child is established [44]. This phenomenon is expected to diminish as the immunological health status of the mothers improves with the use of antiretrovirals. A certain number of immune abnormalities intrinsic to the child have also been described in studies of limited numbers of participants, with sometimes contradictory results, making it impossible to affirm their veracity [36]. Specific humoral responses after vaccination are normal, testifying to an efficient collaboration between T and B lymphocytes and the integrity of the immune system of HIV exposed-uninfected children [44]. Regardless of the cause, the increased risk of infections has led to an enhanced vaccination program in exposed, uninfected children.

### Long-term toxicity

The long-term toxicity is much more difficult to identify and evaluate. The follow-up of uninfected children beyond the period of diagnostic certainty of non-infection (6 months) is particularly difficult to organize. The identification of rare events and their comparison with the incidence observed in the general population is a process for which the complexity is often underestimated in the evaluation of the toxicity profiles of the molecules. An increased risk of cancer associated with *in utero* exposure to didanosine was thus demonstrated several years after its use by cross-referencing data from a large cohort of children born to HIV-positive mothers and a national cancer registry [45]. Fortunately, didanosine – a genotoxic nucleoside analog – was very little used by pregnant women during its period of commercialization, which is now over. Other molecules of the same class do not appear to be associated with such a risk, despite a proven *in vitro* genotoxicity profile [46]. Given the difficulties of long-term and large-scale monitoring of exposed non-infected children, the search for a “biological signature” following maternal treatment may be justified. Such biological signatures could be used as a warning signal to target potential clinical problems and for the comparison of molecules.

## Conclusion

Prophylaxis of mother-to-child transmission of HIV with antiretrovirals has been extraordinarily successful. The period of pregnancy and breastfeeding is usually accompanied by the very careful monitoring of treatment by the woman, motivated by the protection of her child. With continued efforts in screening and the organization of care and treatment of pregnant women, it is not unreasonable to expect that pediatric AIDS may virtually disappear and that children will only become infected in rare cases of failure of care and not because of an uncontrollable epidemic.
